# Effect of a three-years preventive medicine vocational education program on county-level healthcare workforce development in China: a cross-sectional study

**DOI:** 10.1186/s12909-025-07095-w

**Published:** 2025-04-11

**Authors:** Hong Li, Chu Chen, Aizhen Chen, Qi Lin, Dan Li, Mingjun Chen, Nengfeng Xu, Hailin Zhuang

**Affiliations:** 1 School of Public Health and Health Management, Fujian Health College, Fuzhou, 350101 Fujian China; 2 School of Health Management, Fujian Medical University, Fuzhou, 350101 Fujian China

**Keywords:** Preventive medicine education, County-Level healthcare, Vocational training, Regional healthcare disparities, Supply-demand alignment, Workforce development

## Abstract

**Background:**

This study aims to analyze the demand and supply of these professionals within healthcare institutions at the county level after the Chinese government launched a three-year vocational education program of preventive medicine in 2016.

**Methods:**

A national cross-sectional design and multistage cluster sampling method were employed for this study. At the county level, a total of 132 Centers for Disease Control and Prevention (CDCs), 346 medical institutions, 20 medical colleges and 1,083 graduate students were selected. Self-designed questionnaires were used to assess the demand and supply of these professionals. Descriptive statistics were applied to describe key data features.

**Results:**

The county-level CDCs and medical institutions required an average annual total of 15,007 preventive medicine professionals. However, vocational colleges have only enrolled 2,025 of these students per year. Moreover, approximately two-thirds of the provinces continued to face significant demand for preventive medicine professionals, Additionally, medical institutions prioritized clinical skills while the CDCs emphasized preventive expertise as essential qualities for preventive medicine professionals. The requirement for personal qualities were consistently of the utmost importance in healthcare institutions. Furthermore, the self-assessments conducted among graduate students in medical institutions have revealed a relative insufficiency of their professional skills.

**Conclusions:**

There was a general shortage of professionals in vocational preventive medicine education in China; however, certain provinces exhibited a surplus supply relative to demand, and prospective graduates primarily secure employment within medical institutions. The enhancement and refinement of professional skills are imperative in order to align with the specific demands of the preventive medicine position.

**Supplementary Information:**

The online version contains supplementary material available at 10.1186/s12909-025-07095-w.

## Background

Regardless of whether a developed or developing country, primary healthcare at the community level faces immense pressure and necessitates the provision of tailored public health services [1–3]. The COVID-19 pandemic, along with other chronic diseases and public health issues, has placed significant burdens on individuals and societies [4, 5]. To effectively address these challenges, preventive medicine plays an indispensable role with its primary objective being the eradication of diseases by preventing their occurrence or mitigating complications after onset. However, many countries were grappling with shortages of preventive medicine professionals [6–8], a situation starkly highlighted and exacerbated by the COVID-19 pandemic [8, 9]. The primary strategy to address the scarcity of primary preventive medicine professionals lies in expanding the pool of well-educated individuals and enhancing the quality of education.

Training systems for preventive medicine professionals have been implemented worldwide. Public health education includes formal higher education and short- and medium-term training programs [10]. In particular, it encompassed vocational education (equivalent to associate’s degree level), undergraduate programs, graduate programs at the master’s level, and doctoral programs [11–14]. For instance, in the United States, a public health college vocational program usually lasts for 2 years, while an undergraduate program lasts 4 years [15–17]. Pakistan has a similar system, with four years of undergraduate, two years of master’s, and three years of doctoral program [18]. Short- and medium-term training also plays an important role in public health education. Such training programs typically span duration of 6 to 12 months in the United Kingdom [19]. Nevertheless, vocational education in public health with a duration of two years or less is often characterized by insufficient knowledge and skills of clinical medicine due to its brevity. Furthermore, the curriculum of public health for vocational education was predominantly confined to statistics, epidemiology, health promotion, and health policy [11].

An enriched and comprehensive curriculum in vocational education for preventive medicine at the county level is imperative. Taking China as an example, there were currently over 100 universities offering majors in preventive medicine, with an annual intake of approximately 10,000 students [20]. However, a shortage persisted, particularly in primary care systems and remote mountainous areas where preventive medicine physicians were lacking. The main challenges included inadequate supply of personnel, lack of specialization, an aging workforce, and the outflow of talent [21]. These systemic challenges stem from fundamental structural characteristics of China’s public health system. As government-funded public goods, primary public health services rely heavily on preventive medicine graduates as service providers. At county-level medical institutions, chronic staffing shortages have compelled clinical practitioners and nursing staff to assume dual roles-fulfilling core public health responsibilities while simultaneously providing revenue-generating clinical services. This role conflation has resulted in compromised specialization and diminished quality of public health services. Compounding these issues is the reluctance of bachelor’s degree holders and higher-qualified professionals to serve in county level or rural positions [22, 23]. To address these challenges, the Chinese government initiated a three-year vocational education program for preventive medicine in 2016 which resulted in 66 institutions offering this program by 2023. Unlike higher education programs such as undergraduate degrees or above, the primary aim of this level of education was to cater to the requirements of the county-level healthcare system. Consequently, vocational education frequently collaborated closely with CDCs and medical institutions to promptly tackle issues pertaining to public health at the county level. As a result, Most of the Graduates from these vocational education programs found employment of CDCs and medical institutions at the county level.

The vocational colleges in China have established a teaching alliance comprising seasoned educators and experts from CDCs, harmonized the draft of countrywide teaching standards, and implemented a three-stage teaching framework encompassing general literacy, fundamentals of medicine, clinical medicine, and specialized courses. However, the course schedules varied among different colleges [24]. Moreover, the COVID-19 pandemic has underscored the imperative of integrating preventive medicine and clinical medicine into primary healthcare services at the county level [8]. Hence, it is imperative to conduct a nationwide investigation on the supply and demand situation as well as job competency for professionals in preventive medicine professionals within vocational education. Therefore, it is crucial to analyze regional disparities in supply and demand within densely populated countries and formulate strategies to address these challenges.

## Methods

### Study design and sampling

Our study was a cross-sectional analysis of a nationwide survey on the supply and demand of preventive medicine students in China. A multistage cluster sampling method was used to recruit the study population from May to September 2023.

For the demand assessment, we employed a multi-stage selection process. In the first stage, we selected 13 provinces and two directly administered municipalities across Eastern, Central, and Western China, based on economic level and geographical location. In the second stage, we selected three cities within each selected province or municipality, also considering economic level (high, medium, and low) and geographical location (coastal and inland). In the third stage, local CDCs and medical institutions, including community health service centers, township health clinics, and county hospitals, identified one district and three counties within each city based on the urban-rural distribution. Finally, we invited the heads of the CDCs and medical institutions to complete the questionnaire. (see Supplementary Fig. 1).

For the supply assessment, the first stage was identical to the demand assessment. In the second stage, In the second stage, we selected colleges that had more than 30 graduates from the chosen provinces and municipalities. In the third stage, we invited the principals of colleges and graduate students from each selected college to participate (see Supplementary Fig. 2).

Overall, this study involved 132 county CDCs, 346 medical institutions, 20 medical colleges, and 1,083 preventive medicine graduate students working in CDCs and medical institutions.

### Data collection

The study collected data using self-designed questionnaires distributed via Questionnaire Star, an online survey platform accessible through mobile devices. Participants accessed the survey by scanning QR codes shared through WeChat. The survey remained open from the distribution of the survey invitation until September 30, 2023.

### Questionnaire

We utilized a self-designed questionnaire developed by a panel of experts in medical education, public health, preventive medicine, and biostatistics. The healthcare institution demand questionnaire consisted of four parts: the first part collected basic information of current employees, including total number of employees, age, gender, and educational background; the second part examined the implementation of recruitment plans, including historical planned and actual recruitment numbers for preventive medicine professionals from 2020 to 2022, as well as the actual demand numbers for 2023–2025; the third part assessed professional talent requirements, spanning five dimensions—personal qualities, foundation courses, specialized courses, clinical medicine courses, professional Skills—across a total of 52 items which were listed in the Supplementary Questionnaire, each rated on a five-point Likert scale ranging from “no requirement” to " high level of necessity”; and the fourth part included three semi-open questions aimed at understanding the strengths and weaknesses of preventive medicine professionals.

For the supply assessment, two distinct questionnaires were used: one targeting medical schools and the other targeting graduates. The medical school questionnaire collected information on the enrolment numbers of preventive medicine students, the courses offered, and students’ performance in professional qualification examinations. The graduate questionnaire covered three main aspects: personal basic information (e.g., gender, alma mater), current job position, and self-assessment of how well their education prepared them in terms of personal qualities, professional knowledge, skills, and overall competencies relative to their job requirements. Similar to the demand-side questionnaire, it included 52 items across the same four dimensions, each rated on a five-point Likert scale ranging from “extremely inadequate” to “very adequate”.

### Curriculum

The three-year preventive medicine curriculum in China is generally semester-based, with each semester focusing on developing specific competencies and progressively deepening medical knowledge (see Supplementary Table 1). This structured approach ensures a comprehensive understanding of Preventive Medicine. In the first semester of the first year, students focus on developing personal qualities such as Moral Qualities, Ideals and Beliefs, and Life Education, etc. In the second semester of the first year, students study foundational courses including Microbiology and Immunology, Diagnostic Medicine, Pharmacology, etc. In the first semester of the second year, students delve into clinical medicine courses such as Internal Medicine, Emergency Medicine, and Infectious Diseases, etc. In the second semester of the second year, students focus on specialized courses in preventive medicine, including Field Epidemiology, Health Statistics Practice, and Occupational Health and Occupational Medicine, etc. In the third year, students gain practical experience through internships in county-level medical institutions or CDCs.

### Definition of variables

#### Demand variables

we used the “planned recruitment numbers for the past three years (2020–2022), actual recruitment numbers” and “planned demand numbers for the next three years (2023–2025)” as the quantitative of demand variables, and used the score of the required job competencies for preventive medicine, including personal qualities, professional knowledge, professional skills, and comprehensive abilities, as specified in the questionnaire, were used to reflect the quality of demand.

#### Supply variables

For the quantitative aspect of supply, we selected the historical enrollment numbers of preventive medicine students from medical schools as the variable indicating supply volume. For the qualitative aspect, we utilized graduates’ assessments of their education in terms of personal qualities, professional knowledge, professional skills, and comprehensive abilities in relation to job requirements as the variable representing the quality of educational training provided by the institutions.

#### Assessment of other variables

In addition to other variables, for example, number of populations for the province was sourced from the China Statistical Yearbook [25].

### Statistical analysis

Statistical analysis was conducted using Stata version 17. Descriptive statistics, including frequencies, percentages, means, and standard deviations, were used to summarize categorical and continuous variables, respectively.

Due to the non-normal distribution of data, the Kruskal-Wallis test was employed to compare institutional requirements and graduates’ self-assessment scores. The Wilcoxon test was utilized for pairwise comparisons between dimensions scores.

## Results

### Demographic characteristics of demand institutions

A total of 478 healthcare institutions were surveyed on the demand side, including 132 CDCs, which consist of 69 district and 63 county CDCs, and 346 county-level medical institutions, comprising 187 urban and 159 rural facilities. Among the CDCs, most were in the western region (34.8%). These CDCs employed a median of 61 staff members, with 12 positions specifically dedicated to preventive medicine roles. The educational background of the staff in these positions was predominantly at the bachelor’s level, followed by associate degrees, with the majority holding junior professional titles. In the case of medical institutions, there was a median of 61 staff members, but only 1 position designated for preventive medicine roles. The academic qualifications in these roles were primarily at the associate degree level, and similar to CDCs, the majority held junior professional titles. Detailed information is provided in Table 1.

**Table 1:** Demographic characteristics of demand institution (*n* = 478)

Variables	CDCs *N* = 132 *n*(%)	Medical institution *N* = 346 *n*(%)
Area		
Eastern China	41(31.06)	115(33.24)
Central China	45(34.09)	108(31.21)
Western China	46(34.84)	123(35.55)
Total number of staff P_50_(P_25_, P_75_)	61 (43, 95)	61 (31, 117)
Total preventive medicine staff P_50_(P_25_, P_75_)	12 (6, 22)	1 (0, 2)
Education		
Associate	11.76 ± 13.58	6.08 ± 11.70
Bachelor	20.61 ± 18.49	4.82 ± 10.30
Graduate	2.55 ± 7.31	0.21 ± 1.08
Title		
Senior	1.42 ± 2.19	0.17 ± 0.71
Associate senior	5.18 ± 5.51	0.86 ± 1.86
Intermediate	10.33 ± 8.77	2.65 ± 4.72
Junior	13.02 ± 12.36	5.71 ± 11.48
None	7.49 ± 10.97	1.94 ± 5.35

### Demographic characteristics of graduates

The survey on the demand side included 20 medical vocational colleges, distributed as follows: 8 in Eastern China, 6 in Central China, and 6 in Western China. Data were collected from a total of 1,083 graduates from these colleges, among whom 311 were working in CDCs and 772 were working in medical institutions. Of the surveyed graduates, 72.48% were female. Graduates who had completed their education between 3 and 5 years ago were 57.89% of the total sample (see Table 2).

**Table 2:** Demographic characteristics of graduates (*n* = 1,083)

Variables	*n* (%)
Gender	
Male	298(27.52)
Female	785(72.48)
Employing institutions	
CDCs	311(28.72)
Medical institutions	772(71.28)
Years since graduation	
<3 years	430(39.71)
3–5 years	627(57.89)
>5 years	26(2.40)

### Shortage and needs in CDCs and medical institutions

Table 3 presents the planned recruitment numbers and actual recruitment fulfillment rates for CDCs and medical institutions in Eastern, Central, and Western China from 2020 to 2022. The recruitment implementation rate of CDCs in the western region surpassed that of the central and eastern regions, with Qinghai and Yunnan achieving a perfect 100.00% while Guangxi reached an impressive 92.59%. However, there were no plans for recruitment in municipalities and in eastern region such as Jiangsu. Meanwhile, the recruitment implementation rate of medical institutions in the western region was comparatively lower than that in the eastern region, as indicated in Table 3.

**Table 3:** Recruitment of preventive medicine personnel (2020–2022)

Area	Province	CDCs		Medical institutions	
		Planned recruitment	Recruitment Implementation Rate (%)	Planned recruitment	Recruitment Implementation Rate (%)
Eastern	Tianjin	0	0/0	3	66.67
	Guangdong	663	41.18	1966	37.50
	Fujian	225	74.51	265	59.26
	Shandong	491	66.07	181	68.18
	Jiangsu	0	0/0	2597	82.05
Central	Henan	229	10.53	446	21.05
	Anhui	300	77.78	268	0.00
	Hunan	247	35.00	52	42.86
	Jilin	311	70.79	696	87.76
Western	Guizhou	83	88.24	582	7.41
	Guangxi	1376	92.59	962	54.55
	Sichuan	210	60.00	291	14.29
	Chongqing	0	0/0	87	28.57
	Qinghai	139	100.00	84	25.00
	Yunnan	47	100.00	357	39.29
Whole		288	70.65	589	51.87

Notes: We made proportional adjustments by dividing the total population by the population served by the sample healthcare settings. Number of Specialized Preventive Medicine Personnel Planned to be Recruited for Each Province = Number of Personnel Planned to be Recruited by Each Unit (2020–2022) * (Permanent Population of Each Province / Permanent Population Served by Surveyed Units); Recruitment Implementation Rate = Number of Specialized Preventive Medicine Personnel Actually Recruited by Units / Number of Specialized Preventive Medicine Personnel Planned to be Recruited * 100%

Table 4 shows that there remains a significant demand for professionals in preventive medicine during 2023–2025, with the CDCs requiring 2,095 individuals and medical institutions needing 12,912 individuals. On a provincial level, medical institutions continued to exhibit a high demand, yet in certain provinces, the demand from the CDCs was zero. These provinces include Tianjin, Jiangsu, Guangxi, Guizhou, Chongqing and Yunnan.

**Table 4:** Average annual demand of preventive medicine personnel (2023–2025)

Area	Province	CDCs	Medical institutions
Eastern	Tianjin	0	3
	Guangdong	650	2436
	Fujian	143	328
	Shandong	292	363
	Jiangsu	0	3951
Central	Henan	277	398
	Anhui	140	856
	Hunan	227	124
	Jilin	189	244
Western	Guangxi	0	1840
	Guizhou	0	334
	Sichuan	21	1364
	Chongqing	0	90
	Qinghai	156	103
	Yunnan	0	476
Whole		2095	12,912

Notes: We made proportional adjustments by dividing the total population by the population served by the sample healthcare settings. Number of Specialized Preventive Medicine Personnel Demand for Each Province = Number of Personnel Demand by Each Unit (2023–2025) * (Permanent Population of Each Province / Permanent Population Served by Surveyed Units).

**Fig. 1:**
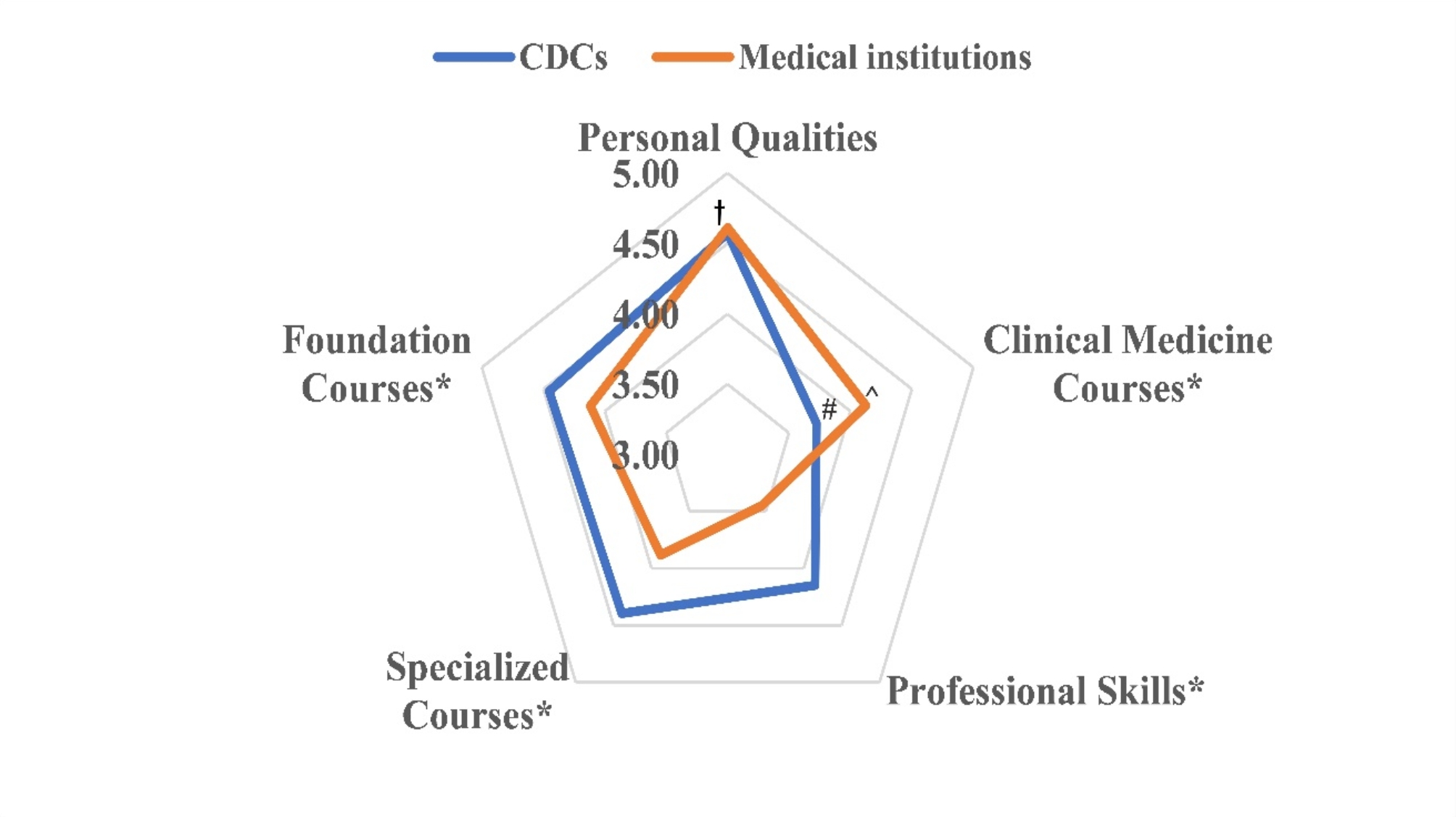
Requirements for preventive medicine professionals in CDCs and medical institutions. * *p* < 0.05 CDCs vs. medical institutions. **#***p* < 0.05 clinical medicine courses vs. personal qualities, specialized courses, professional skills and foundation courses in CDCs. ^ *p* < 0.05 clinical medicine courses vs. specialized courses or professional skills in medical institutions. † *p* < 0.05 personal qualities vs. clinical medicine courses, specialized courses (only in medical institutions), professional skills and foundation courses (only in medical institutions). These five dimensions were assessed using a Likert scale ranging from “no requirement " to " high level of necessity”, with a rating of 5 indicating a high level of necessity, 4 indicating a relatively high level of necessity, 3 indicating moderate necessity, 2 indicating lower necessity, and 1 indicating no requirement

There were significant differences in the qualifications required for preventive medicine professionals between CDCs and medical institutions. As shown in Fig. 1, medical institutions placed a greater emphasis on clinical medicine courses (*p* < 0.05). In contrast, CDCs prioritized foundational courses (*p* = 0.05) and specialized courses (*p* < 0.05) and professional skills (*p* < 0.05). For the personal qualities no significant statistical differences between CDCs and medical institutions were observed (*P* = 0.05). Among the five dimensions of CDCs, clinical medicine courses exhibit the lowest demand score (*p* < 0.05). In medical institutions, the demand score for clinical medicine courses surpasses that of specialized courses and professional skills (*p* < 0.05). Irrespective of whether it pertains to CDCs or medical institutions, personal qualities demonstrate the highest demand score and exceed most of other dimensions(*p* < 0.05), except for specialized courses and foundation courses in CDCs.

### Supply of preventive medicine professionals

Table 5 demonstrates the average annual enrollment of preventive medicine students in vocational colleges across the eastern, central, and western China. Over the past three years (2020–2022), Shandong Province had the highest with 355, followed by Hunan with 346, Jiangsu with 302, and Chongqing with 179. Guangxi Province had the lowest, with only 20.

**Table 5:** Average annual enrollment of preventive medicine students in vocational colleges (2020–2022)

Area	Province	Average annual enrollment numbers
Eastern	Tianjin	57
	Guangdong	113
	Fujian	119
	Shandong	355
	Jiangsu	302
Central	Henan	61
	Anhui	126
	Hunan	346
	Jilin	59
Western	Guangxi	20
	Guizhou	114
	Sichuan	36
	Chongqing	179
	Qinghai	33
	Yunnan	105
Whole		2025

Notes: The enrollment numbers of each province is derived by multiplying the average number of admissions in surveyed colleges within that province with the total count of colleges admitting students therein

### Matching of supply and demand of preventive medicine professionals

#### Quantity matching

**Fig. 2:**
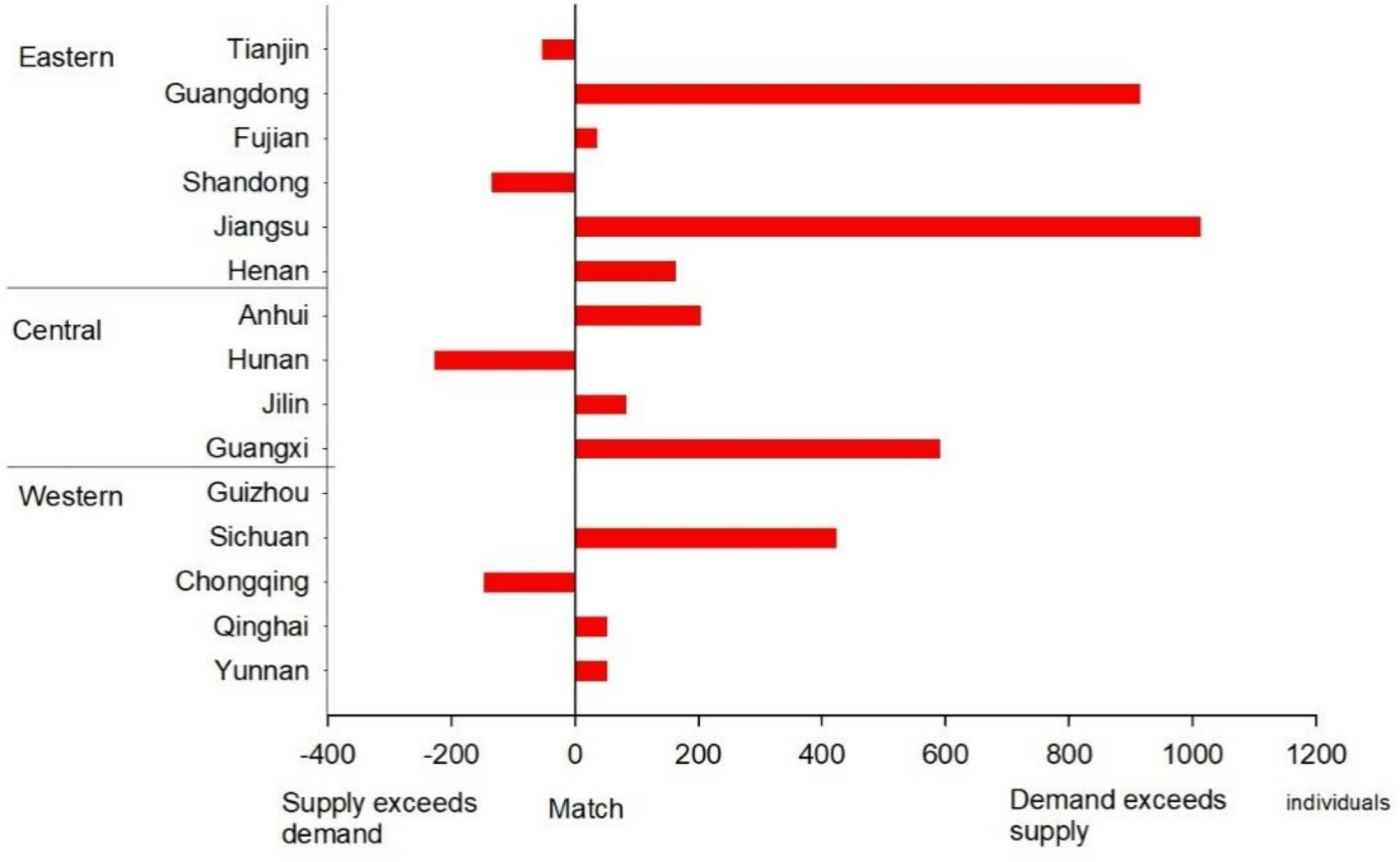
Alignment of supply and demand for preventive medicine professionals

Notes: Alignment of supply and demand: the difference between the planned recruitment numbers for preventive medicine professionals for 2023–2025 and the average annual enrollment numbers of college from 2020 to 2022 across Eastern, Central, and Western China.

Figure 2 illustrates the alignment between the planned recruitment numbers for preventive medicine professionals for 2023–2025 and the average annual enrollment numbers of college from 2020 to 2022 across Eastern, Central, and Western China. The data revealed that approximately two-thirds of the provinces continued to experience significant demand for preventive medicine professionals. Notably, the supply shortage was most pronounced in Jiangsu and Guangdong. In contrast, provinces such as Hunan, Chongqing, Shandong and Tianjin, demonstrated an oversupply of graduates relative to the planned recruitment numbers. These mismatches underscored the need for region-specific adjustments to better balance the supply of graduates with the healthcare workforce.

#### Quality matching

**Fig. 3:**
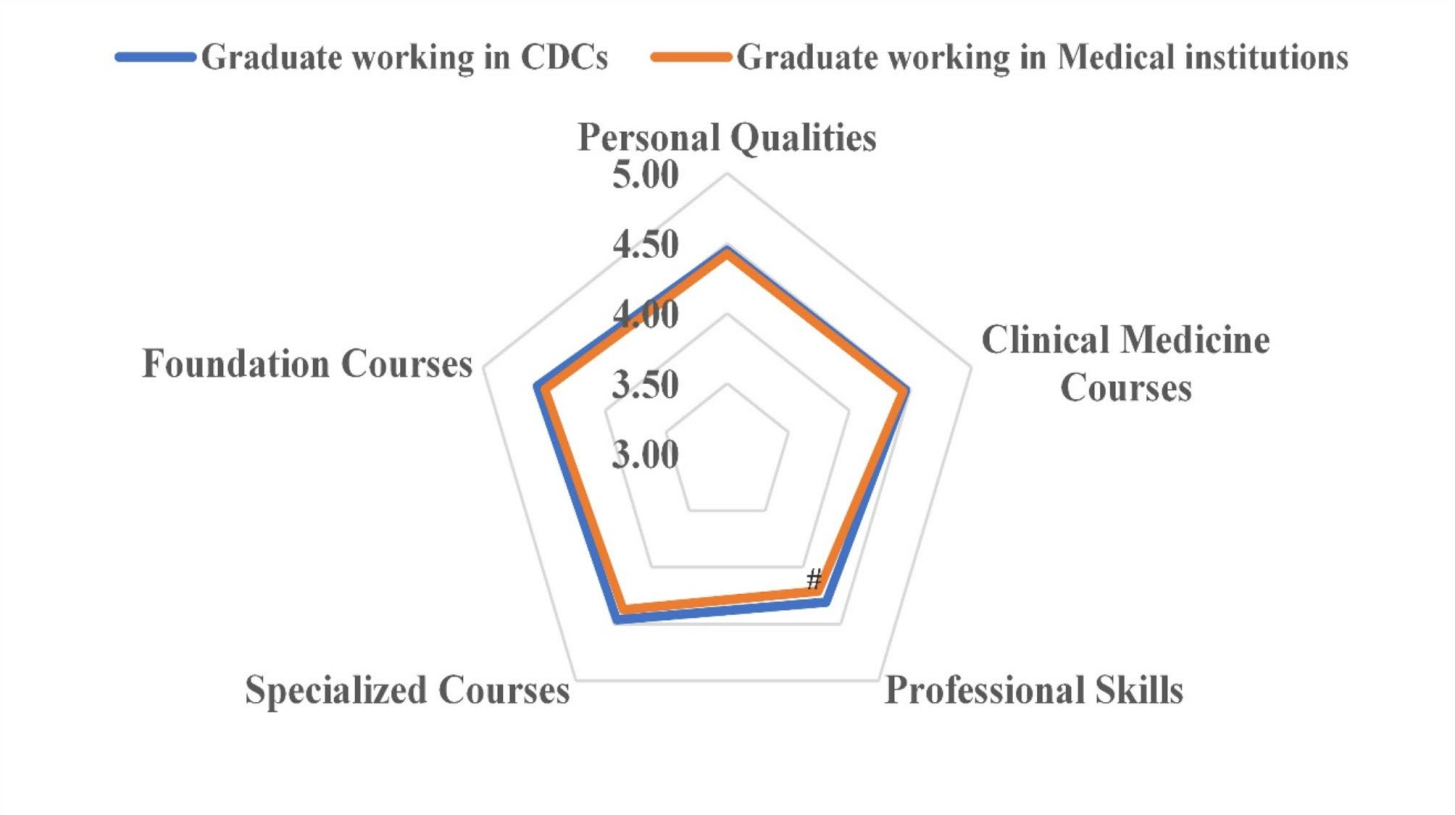
Self-assessment of preventive medicine graduates in CDCs and medical institutions. # (*p* < 0.05) professional skills vs. the other four dimensions in medical institutions. The five dimensions are assessed using a five-point Likert scale, ranging from “extremely inadequate” to “very adequate”; with 5 points assigned to “very adequate”, 4 points to “adequate”, 3 points to “essentially adequate”, 2 points to “not adequate”, and 1 points to “extremely inadequate”

Figure 3 illustrates no statistically significant differences in the self-assessment scores were observed between graduates from CDCs and medical institutions. However, when considering different organizations, there were no overall statistical significances in the differences of self-evaluation scores among CDCs graduates across the five dimensions (*p* = 0.40). In contrast, a statistically significant difference across the five dimensions existed among graduates from medical institutions (*p* = 0.01), with their professional skills score being significantly lower than personal qualities (*p* = 0.02), clinical medicine courses (*p* = 0.03), specialized courses (*p* = 0.03), and foundation courses (*p* < 0.01). This suggested that graduates in medical institutions expressed relatively lower satisfaction with their skills.

## Discussion

To the best of our knowledge, this study is the first nationwide survey in China that focuses on preventive medicine professionals from vocational colleges. The findings can help future vocational schools better manage the enrollment scale and course offerings for preventive medicine programs. We found that the demand for preventive medicine professionals in county-level CDCs and medical institutions will predominantly exceed supply in the next three years (2023–2025). This shortage aligns with previous research findings [26–28]. The demand for preventive medicine professionals in county-level CDCs and medical institutions was examined separately in our survey, revealing a significant disparity. Specifically, the demand within medical institutions was found to be six times higher than that of CDCs. This suggests an increasing trend towards career opportunities for graduates in preventive medicine within county-level medical institutions. The primary reason for the aforementioned phenomenon can be attributed to the imbalanced distribution of medical institutions and CDCs in China. As of 2021, there were a total of 10,122 community health service centers, 34,943 township hospitals, and 3,376 CDCs in China. The combined number of the first two types of institutions is approximately 13.3 times that of the number of CDCs. The total number of physicians (licensed physicians and assistant licensed physicians) in community health service centers and township hospitals is 2,478,126, while the corresponding number in CDCs is 139,247, which is about 17.8 times higher [25]. Furthermore, as non-profit organizations primarily funded by the government, local governments in China have mandated county-level CDCs to recruit graduates with a bachelor’s degree or higher in preventive medicine, aiming to enhance the development of human resources, particularly in developed regions. This has significantly limited entry opportunities for graduates from vocational education programs in preventive medicine [29–31]. According to our survey, the annual planned recruitment numbers (2020–2022) for county-level CDCs in municipalities or developed areas such as Tianjin, Jiangsu, and Chongqing were nearly zero, and from 2023 to 2025 more county-level CDCs did not plan to recruit the graduates of this level. Given the current expansion enrollment scale for undergraduate and above preventive medicine, this trend may become even more pronounced.

We also discovered regional differences in demand between the central, eastern, and western areas, which is consistent with previous studies [32]. The significance of these inequalities lies in the disparities observed in regional levels of public health services [33]. We found that from 2023 to 2025, the demand in the eastern and central regions was high, while the supply was insufficient. Conversely, the western region had an adequate supply, which might be related to the high recruitment implementation rate in the western CDCs from 2020 to 2022. However, it was also evident that the recruitment implementation rate for medical institutions in the western region was significantly lower than that in the central and eastern regions. The occurrence of this phenomenon can be ascribed to the geographical distribution of county-level medical institutions in economically underdeveloped areas within western China, which is further complicated by the presence of diverse ethnic groups, linguistic variations, and cultural customs [34–36]. Consequently, there is a pressing need for local expertise in preventive medicine; however, cultivating such professionals remains a significant challenge.

Our survey has observed that graduates generally met the job requirements within the current curriculum system. However, given the disparities in course requirements between county-level CDCs and medical institutions, it is imperative to make reasonable adjustments to course content within a focused scope. First, we have observed a distinct emphasis on personal qualities in both types of institutions. In the context of county-level preventive medicine work, effective communication and interpersonal skills are deemed essential prerequisites. Consequently, it is imperative for educational institutions to acknowledge the significance of incorporating courses that cater to these domains. Secondly, our survey revealed that clinical medical courses were consistently ranked higher by medical institutions compared to CDCs. However, many higher education institutions in China tend to overlook clinical medical education and skills training when cultivating talents in preventive medicine across various academic levels (from associate degree to doctorate) [37]. This is primarily attributed to the fact that graduates of preventive medicine majors are not authorized to prescribe medications as public health physicians [38]. Furthermore, the current job requirements for county-level CDCs positions did not place sufficient emphasis on clinical knowledge. Nevertheless, the positions in county-level medical institutions required expertise related to child health management, maternal health management, etc., while CDCs positions associated with infectious disease prevention and control, HIV/STD prevention and control as well as occupational health are closely intertwined with clinical knowledge [39]. Considering the growing demand for professionals in preventive medicine at county-level medical institutions, colleges should not only prioritize public health but also emphasize cultivating of clinical expertise. The outbreak of the COVID-19 pandemic has highlighted the fragmentation between clinical care and preventive medicine in China [8]. Therefore, promoting the integration of clinical care and preventive medicine is imperative for enhancing disease prevention and control.

Interestingly, our survey findings indicated that the graduates enrolled in county-level medical institutions exhibited satisfaction with their clinical courses. Two primary factors may account for this finding. Firstly, vocational colleges have established closer collaborations with county-level medical institutions, thereby recognizing the necessity of clinical skills and reinforcing them accordingly. Key measures included implementing clinical training within teaching medical institutions, integrating learning-teaching-internship approaches, and engaging additional clinicians in clinical instruction. Secondly, it was plausible that these students were not assigned to positions entailing higher levels of clinical demand at medical facilities such as immunization planning or outpatient services [40]. In contrast to this observation, graduates expressed discontentment regarding their skill training. Despite offering a wide range of skill options, graduates satisfaction remained low primarily due to inconsistent skill training standards across different colleges and the fact that current professional positions require advanced skills beyond what was covered in textbooks. In China’s counties, through health system reforms, compact county medical community have become an important vehicle for integrating clinical care and preventive medicine [41, 42]. Consequently, the need for preventive medicine graduates to have clinical medical skills in medical institutions has become more apparent. It is noteworthy that, in comparison to other dimensions, both CDCs and graduates from medical institutions exhibited the lowest level of satisfaction with their professional skills training received during their academic years, particularly among those who have graduated from medical institutions. Given the distinct disparities in required professional skills for CDC and medical institution positions, it becomes imperative to conduct comprehensive research on the actual industry-specific professional skills demanded by these roles and subsequently adopt teaching methodologies that are aligned with these requirements.

Our survey has important implications for the education and training of preventive medicine professionals. The findings indicated a considerable demand for preventive medicine specialists at the county level, both in CDCs and medical institutions. In the context of China’s educational system, vocational colleges predominantly prepared preventive medicine students for employment at the county level. Therefore, it is advisable to substantially increase the enrollment and training of students in vocational programs for preventive medicine. However, it is crucial to note that in the western regions of China, the current enrollment scale for preventive medicine students already surpassed the existing demand. As a result, institutions in these regions should consider reducing enrollment scale in the future. Moreover, in terms of curriculum design, there should be a concerted effort to integrate clinical and preventive medicine education to better equip students for future employment opportunities.

Our study’s assessment of the supply and demand of preventive medicine professionals primarily depended on self-reported data, lacking objective performance indicators such as the actual number of registered physicians in the health information system, the actual number of graduates from educational institutions, or the third-party data validation. Additionally, it may be constrained by the anchoring effect and recall bias inherent in self-reports. While the self-report method is commonly used in health workforce research, cognitive biases can introduce uncertainty into the evaluation of the supply-demand relationship. Given these limitations, future research should aim to integrate multiple methods, such as the Skill Matrix Matching Method and Big data Semantic Analysis, for more comprehensive predictions.

## Conclusion

Our research indicates a significant demand for preventive medicine professionals at county-level CDCs and medical institutions. Currently, there is a shortage in supply in the eastern and central regions of China, while the western regions show an oversupply of graduates. To better align with the employment needs and enhance the career prospects of students, future curriculum development should integrate specific courses with clinical medicine training. The enhancement and refinement of professional skills are imperative in order to align with the specific demands of the preventive medicine positions.

## Electronic supplementary material

Below is the link to the electronic supplementary material.

Supplementary Material 1

Supplementary Material 2

Supplementary Material 3

Supplementary Material 4

## Data Availability

These datasets can be obtained from the corresponding author upon reasonable request, pending approval from the National Disease Control and Prevention Administration of China.
