# Supplementary material for: Effect of a three-years preventive medicine vocational education program on county-level healthcare workforce development in China: a cross-sectional study
Source: BMC Med Educ. 2025 Apr 11;25:522. doi: 10.1186/s12909-025-07095-w (PMC11992889; doi:10.1186/s12909-025-07095-w)
Supplement: Supplementary file 2 — Supplementary Material 2 [file 12909_2025_7095_MOESM2_ESM.pdf]

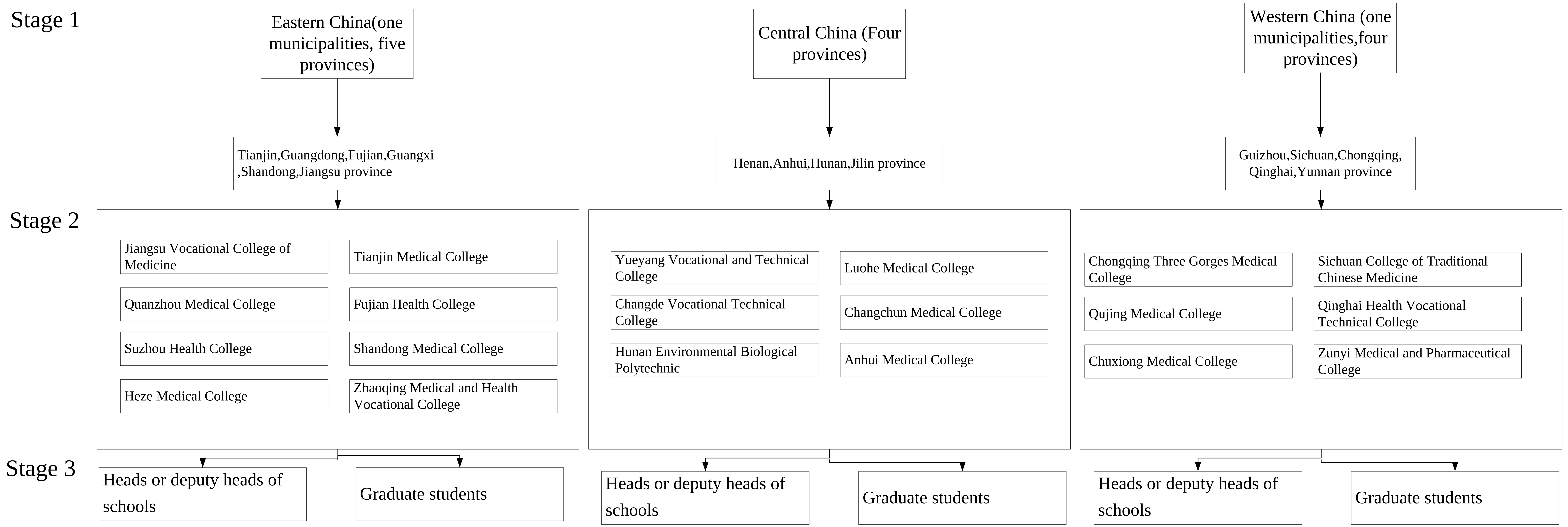

Figure 2 colleges and graduates included
