# Supplementary material for: Effect of a three-years preventive medicine vocational education program on county-level healthcare workforce development in China: a cross-sectional study
Source: BMC Med Educ. 2025 Apr 11;25:522. doi: 10.1186/s12909-025-07095-w (PMC11992889; doi:10.1186/s12909-025-07095-w)
Supplement: Supplementary file 3 — Supplementary Material 3 [file 12909_2025_7095_MOESM3_ESM.pdf]

Questionnaire 1

Demand of Professionals of Three Years of Vocational Education in Preventive Medicine within County Healthcare Institutions

1. Name of the institution: ______; Address: Province/ City/ County /(District)________

2. Resident population number served by your instruction: ______.

3. Geneal Staffing status within the institution

If CDCs	If Medical institutions
Total Number of Staff in Your institution: ______,<br>Including Health Technicians: ______.<br>Set Up __, Actual _;<br>Among Them:<br>① Graduates of Preventive Medicine: ______,<br>② Graduates from Other Medical Majors Who Have Received Specialized Training: ______,<br>③ Graduates from Other Medical Majors Without Specialized Training: ______.	Total Number of Staff in Your Institution ______,<br>Including Health Technicians: ______.<br>Among Them:<br>① Graduates of Preventive Medicine: ______,<br>② Graduates from Other Medical Majors Who Have Received Specialized Training: ______,<br>③ Graduates from Other Medical Majors Without Specialized Training: ______.

4. The number of Gender composition of preventive medicine professionals:
Number Female ____; Number of Male ______,

5. Age composition of preventive medicine professionals:
① Number of professionals <30 years old ______
② Number of professionals within 30-39 years old ______
③ Number of professionals within 40-49 years old ______
④ Number of professionals ≥50 years old ______

6. The number of preventive medicine professionals with different educational backgrounds:
① Associate ____ ② Bachelor _____ ③ Graduate _____

7. The number of personnel with different professional titles:
① Senior Title ______ ② Associate Senior Title ______ ③ Intermediate Title ______ ④ Junior Title ______ ⑤ No Title ______

8. Planned recruitment number for preventive medicine professionals in the last 3 years (2020-2022):
①Junior College (Vocational) ____ ② Bachelor's Degree ____ ③ Master's Degree ______

9. Actual Recruitment Number for Preventive Medicine Professionals in the Last 3 Years (2020-

approved by the Institutional Review Board at Fujian Health College

2022):
① Junior College (Vocational) _____ ② Bachelor's Degree _____ ③ Master's Degree ______

10. Employment status and benefits of recruited professionals of 3 years of vocational education in preventive medicine in the last 3 years (2020-2022):

11. Turnover of newly hired preventive medicine professionals in the last 3 years (2020-2022):
① Number of Junior College (Vocational) Professional Resigned ______
② Number of Bachelor's Degree Professional Resigned ______
③ Number of Master's Degree Professional Resigned ______

12. Minimum educational requirement for recruiting preventive medicine graduates in the next 3 years (2023-2025):
① Secondary Vocational Education ② Junior College (Vocational) ③ Bachelor's Degree
④ Master's Degree ⑤ No Plan to Recruit (Skip to Question 14)

13. Potential demand for junior college (vocational) preventive medicine professionals in the next 3 years (2023-2025):

If CDCs	If Medical institutions
Demand of Professionals of Three Years of Vocational Education in Preventive Medicine______;<br>(Full-time counts as 1, part-time counts as 0.5)	Demand of Professionals of Three Years of Vocational Education in Preventive Medicine______;<br>(Full-time counts as 1, part-time counts as 0.5)
Positions Related to Preventive Medicine in<br>① Department of Infectious Disease Control: Set Up __, Actual _;<br>② Department of Immunization Planning: Set Up ___, Actual __;<br>③ Department of HIV/AIDS and STD Control: Set Up ______, Actual ______;<br>④ Department of Tuberculosis Control: Set Up __, Actual ____;<br>⑤ Department of Endemic and Parasitic Disease Control: Set Up ______, Actual ______;<br>⑥ Department of Public Health: Set Up ______, Actual ______;<br>⑦ Department of Health Education: Set Up ___, Actual ______;<br>⑧ Department of Emergency Response: Set Up ___, Actual ____;<br>⑨ Department of Occupational Health: Set Up ____, Actual ____;<br>⑩ Other: Set Up ____, Actual _____;	Positions Related to Preventive Medicine in<br>① Department of Vaccination: Set Up ___, Actual _____;<br>② Department of Child Health Management: Set Up ______, Actual ______;<br>③ Department of Maternal Health Management: Set Up ______, Actual ______;<br>④ Department of Chronic Disease Management: Set Up ______, Actual ______;<br>⑤ Department of Record Management: Set Up, Actual ___;<br>⑥ Department of Health Education: Set Up _, Actual __;<br>⑦ Health Supervision Assistants: Set Up ___, Actual __;<br>⑧ Public Health Information Reporters (Infectious Disease Reporters): Set Up ______, Actual ______;<br>⑨ Other: Set Up ______, Actual ______;<br>Among Them:<br>① Graduates of Preventive Medicine: ______,

approved by the Institutional Review Board at Fujian Health College

Among Them:<br>① Graduates of Preventive Medicine: ______,<br>② Graduates from Other Medical Majors Who Have Received Specialized Training: ______,<br>③ Graduates from Other Medical Majors Without Specialized Training: ______.	② Graduates from Other Medical Majors Who Have Received Specialized Training: ______,<br>③ Graduates from Other Medical Majors Without Specialized Training: ______.

14. Does your institution have the qualifications to allow staffs to apply for the Public Health Practitioner (Assistant) Physician Licensing Examination? ① Yes ② No

15. Which certifications provide a greater advantage in employment for the Professionals of three years of vocational education in preventive medicine? (Multiple choices allowed):
①Public Health Practitioner (Assistant) Physician ② Special Equipment Operator Certificate ③ Laboratory Animal Practitioner Certificate ④ PCR Certification ⑤ Others

16. Please evaluate the requirements for professionals of three years of vocational education in preventive medicine at your instruction. Five Levels of Requirements:
⑤ high level of necessity ④ moderate necessity ③ moderate necessity ② lower necessity ① no requirement

Dimension	Course or Skill	Level of Requirement
**Personal Qualities**	Moral qualities	
	Ideals and beliefs	
	Life education	
	Professional competence	
	Humanistic literacy	
	Comprehensive judgment ability	
	Psychological quality	
	Innovation awareness	
	Teamwork awareness	
	Safety awareness	
**Fundamental Courses**	Medical Ethics	
	Computer Applications in Healthcare	
	Microbiology and Immunology	
	Physiology	
	Pharmacology	
	Biochemistry	
	Psychology	
**Clinical Medicine Courses**	Diagnostic Medicine	
	Internal Medicine	
	Surgery	
	Obstetrics and Gynecology	
	Pediatrics	

approved by the Institutional Review Board at Fujian Health College

	Emergency Medicine
	Infectious Diseases
**Specialized Courses**	Epidemiology
	Biostatistics
	Occupational Health and Occupational Medicine
	Environmental Health
	Nutrition and Food Hygiene
	Health Education and Promotion
	Maternal, Child, and Adolescent Health
	Basic Public Health Service Techniques
	Social Medicine
	Health Laws and Regulations
	Integrated Preventive Medicine Skills Practicum
**Professional Skills**	Air Quality Testing Skill
	Water Quality Testing Skill
	Occupational Health Testing Skill
	Food Hygiene Testing Skill
	Child and Adolescent Health Assessment Skill
	Epidemiological Methods and Skills
	Statistical Methods and Skills
	Health Education Skills
	Basic Clinical Skills
	Information Literacy and Professional Resource Use
	Essential Computer Skills
	Literature Search and Review Skills
	Scientific Writing and Research Skills
	Teamwork and Collaboration Skills
	Organizational and Coordination Skills
	Communication and Counseling Skills
	Community Mobilization Skills

17. What types of on-the-job training does your institution provide for professionals of three years of vocational education in preventive medicine?

____

18. What strengths and weaknesses do your institution perceive in professionals of three years of vocational education in preventive medicine?

____

approved by the Institutional Review Board at Fujian Health College

19. Which skills and qualities does your institution believe are most important to strengthen in professionals of three years of vocational education in preventive medicine?

____

This questionnaire is now complete. Thank you very much for your participation!

approved by the Institutional Review Board at Fujian Health College

Questionnaire 2

Survey on the Preventive Medicine Students in Medical Colleges

1. Name of your medical college:________; Address: Province/ City/ County /(District)_______

2. Year your college's preventive medicine program began enrolling students?
① 2015 ② 2016 ③ 2017 ④ 2018 ⑤ 2019 ⑥ 2020

3. Annual enrollment numbers (actual reporting students) and source information for the preventive medicine program at your college
① First year admission, Year:______
Including________ high school graduates and________secondary vocational school graduates
② Second year admission, Year:______
Including________high school graduates and________secondary vocational school graduates
③ Third year admission, Year:______
Including________ high school graduates and________secondary vocational school graduates
④ Fourth year admission, Year:______
Including________ high school graduates and________secondary vocational school graduates
⑤ Fifth year admission, Year:______
Including________ high school graduates and________secondary vocational school graduates
⑥ Sixth year admission, Year:______
Including________ high school graduates and________secondary vocational school graduates
⑦ Seventh year admission, Year:______
Including________ high school graduates and________secondary vocational school graduates

4. Number of full-time preventive medicine faculty______, among whom:
Professors: ________, Associate Professors: ________, Lecturers:________, Assistant Lecturers: ________
Doctoral Graduates: ________, Master's Graduates:________, Bachelor's Degree and Below:_
Dual-Qualified (holding both teaching qualification and Public Health Practitioner license):____
Provincial Teaching Experts:________, Provincial Program Leaders:________, Other Provincial Honor Teachers:________, Industry Part-Time Teachers: _________

5. Overall student satisfaction for your college's preventive medicine program over the past 3 years: ___
① ≥90% ② 85%～90% ③ 80%～85% ④ 75%～80% ⑤ ＜75%

approved by the Institutional Review Board at Furjan Health College

6. What are the main employment destinations for graduates of the preventive medicine program at your college?

① Centers for Disease Control and Prevention ② Health Supervision Institutes ③ Health Education Institutes ④ Community Health Service Centers ⑤ Township Health Clinics ⑥ County-level and Above Hospitals ⑦ Village/Private Health Clinics ⑧ Health Enterprises ⑨ Further Education (Bachelor's Degree) ⑩ Other

7. The total duration of internships at your college is __ weeks, and there are __ different internship arrangements: ① Clinical (__ weeks) + Centers for Disease Control and Prevention (__ weeks) + Community Health Service Centers or Township Health Clinics (__ weeks)

② Clinical (__ weeks) + Health Supervision (__ weeks) + Community Health Service Centers or Township Health Clinics (__ weeks)

③ Clinical (__ weeks) + Other (__ weeks)

approved by the Institutional Review Board at Fujian Health College

8. Please indicate the construction status of the following professional courses at your college by marking "○" in the appropriate options. (Multiple choices allowed)

**Courses**	Nationally Recognized High-Quality Courses	Provincial-Level Recognized High-Quality Courses	School-Level Recognized High-Quality Courses	Online Resources	Socialized Online Resources (e.g., MOOCs)	Full-Time teacher	Part-Time teacher
Medical Ethics							
Computer Applications in Healthcare							
Microbiology and Immunology							
Physiology							
Pharmacology							
Biochemistry							
Psychology							
Diagnostic Medicine							
Internal Medicine							
Surgery							
Obstetrics and Gynecology							
Pediatrics							
Emergency Medicine							
Infectious Diseases							
Epidemiology							

Biostatistics							
Occupational Health and Occupational Medicine							
Environmental Health							
Nutrition and Food Hygiene							
Health Education and Promotion							
Maternal, Child, and Adolescent Health							
Basic Public Health Service Techniques							
Social Medicine							
Health Laws and Regulations							
Integrated Preventive Medicine Skills Practicum							

approved by the Institutional Review Board at Fujian Health College

9. Besides the above professional courses, what other specialized courses does your school offer? Which textbooks are used for these courses?

____

10. Do you think the curriculum structure of your program is reasonable? What aspects need adjustment?

____

11. Please indicate whether the following laboratory (practical training) projects are offered in your college by marking the appropriate options with a "○".

Skill Module	Skills	Offered	Not Offered
**Air Quality Testing**	Measurement of Meteorological Factors		
	Air and Indoor Air Sampling Techniques		
	Measurement of Sulfur Dioxide, Particulates, and Nitrogen Oxides in Air		
	Formaldehyde Concentration Analysis in Air		

**Water Quality Testing**	Water Sample Collection and Pre-Treatment		
	Measurement of Nitrogen Compounds and Arsenic in Water		
	Measurement of Chemical Oxygen Demand and Dissolved Oxygen in Water		
	Measurement of Total Iron, Hardness, and Chlorides in Water		
	Measurement of Active Chlorine in Bleaching Powder and Residual Chlorine in Water		
**Occupational Health Testing**	Biomonitoring of Occupational Chemical Exposures (e.g., Lead, Benzene) in Blood and Urine		
	Measurement of Biomarkers for Effects of Occupational Exposures (e.g., Cholinesterase Activity)		
	Pneumoconiosis Film Reading		
	Measurement of Free Silica Content in Dust		
	Measurement of Total Dust Concentration and Dispersion		
	Measurement of Physical Hazards (Heat, Noise, Vibration, Radiation)		
	On-Site Industrial Hygiene Assessment and Report Writing		
**Food Hygiene Testing**	Measurement of Protein Content in Food		
	Protein Efficiency Ratio		
	Dietary Assessment		
	Measurement of Ascorbic Acid, Total Sugars, and Arsenic in Food		
	Development of Nutrition Plans		
	Nitrite Measurement		
	Measurement of Food Coloring		
	Foodborne Illness Case Analysis		
	Food Safety Supervision Case Discussions		
**Child and Adolescent Health Assessment**	Growth and Development Assessment		
	Investigation and Assessment of Childhood Obesity		
	Classroom Health Assessment		
**Epidemiological Methods and Skills**	Measurement of Disease Frequency		
	Disease Distribution		
	Cross-Sectional, Cohort, and Case-Control Studies		
	Screening Method Evaluation		
	Experimental Epidemiology Methods		
	Bias Identification and Control		
	Causal Inference in Epidemiology		
	Outbreak Investigation		
	Disinfection Techniques		
	Public Health Emergency Response Simulation		
	Chronic Disease Surveillance and Analysis		
	Evaluation of Vaccination Effectiveness		
	Donning and Doffing of Personal Protective Equipment		

approved by the Institutional Review Board at Fujian Health College

	Environmental Epidemiology Data Analysis		
**Statistical Methods and Skills**	Basic Operations of Common Statistical Software		
	Statistical Description of Measurement and Categorical Data		
	Estimation of Population Means and Hypothesis Testing		
	Common Statistical Tests (t-tests, Chi-square tests)		
	Survey and Experimental Design		
	Statistical Tables and Graphs		
**Health Education Skills**	Health Promotion Materials: Organization and Formulation		
	Health Education and Training Methods		
**Basic Clinical Skills**	Physical Examination		
	Cardiopulmonary Resuscitation (CPR)		
	Recognition of Common Chronic Diseases		
	Recognition of Common Infectious Diseases		
	Emergency and First Aid		
**Information Literacy and Professional Resource Use**			
**Computer Skills**			
**Literature Search and Review Skills**			
**Scientific Writing and Research Skills**			
**Teamwork and Collaboration Skills**			
**Organizational and Coordination Skills**			
**Communication and Counseling Skills**			
**Community Mobilization Skills**			

12. Apart from the laboratory (practical training) projects listed above, what other experimental (training) projects does your school offer?

____

13. Does your college provide specialized training for the Public Health Practitioner (Assistant) Physician Licensing Examination? ① Yes ② No

14. Number of 2019 graduates who took the 2020 Public Health Practitioner (Assistant) Physician Licensing Examination: ______, Pass Rate:__________%, including Practical Skills Assessment Pass Rate: __________% and Theory Assessment Pass Rate: __________%.;
Number of 2020 graduates who took the 2020 Public Health Practitioner (Assistant) Physician Licensing Examination: _______, Pass Rate:__________%, including Practical Skills Assessment Pass Rate: __________% and Theory Assessment Pass Rate: __________%;
Number of 2021graduates who took the 2020 Public Health Practitioner (Assistant) Physician Licensing Examination: _______, Pass Rate:__________%, including Practical Skills Assessment Pass Rate: __________% and Theory Assessment Pass Rate: __________%;

15. Besides the Public Health Practitioner Assistant Physician Licensing Examination, what other professional qualification or certification exam training does your school provide for students?

approved by the Institutional Review Board at Fujian Health College

16. Does your school have preventive medicine students in targeted training programs for grassroots positions?
① Yes, targeted training accounts for__________%,
and the designated organizations are: __________.
② No

17. Does your school have preventive medicine students in commissioned training programs?
① Yes, commissioned training accounts for __________%,
and the commissioning organizations are: __________.
② No

This questionnaire is now complete. Thank you very much for your participation!

approved by the Institutional Review Board at Fujian Health College

Questionnaire 3

Survey on Competence of Professionals with Three Years of Vocational Education in Preventive Medicine

1. Your Gender: ① Male ② Female

2. Full Name of Your Vocational College at Graduation:________________

3. Graduation Year:________________

4. Current Employment Status:
① Working in a preventive medicine-related field
② Working in a field unrelated to preventive medicine
③ Pursuing bachelor’s degree
④ Other

5. Current Employer:
① Center for Disease Control and Prevention
② Health Supervision Institute
③ Health Education Institute
④ Community Health Service Center
⑤ Township Health Center
⑥ County-Level or Above Hospital
⑦ Village/Private Health Clinic
⑧ Health and Wellness Enterprise
⑨ Other

6. Location of Current Employer:
Province: ___ City: ___ County/District: ___

7. Is your current workplace location the same as your hometown?
① In a different province/autonomous region/municipality
② In the same province/autonomous region/municipality but a different city/prefecture
③ In the same city/prefecture but a different district
④ In the same district

8. Main Responsibilities in Your Current Role (Select all that apply):
① Immunization Program
② HIV/AIDS and STD Prevention
③ Tuberculosis Prevention
④ Chronic Disease Prevention

approved by the Institutional Review Board at Fujian Health College

⑤ Emergency Response
⑥ Occupational Health
⑦ Public Health
⑧ Child Health Management
⑨ Maternal Health Management
⑩ Vaccination
⑪ Chronic Disease Management
⑫ Health Record Management
⑬ Health Education
⑭ Health and Family Planning Supervision Assistance
⑮ Infectious Disease Supervision
⑯ Medical Institution Supervision
⑰ Hospital Infection Department
⑱ Administrative Management
⑲ Other

9. Employment Type:
① Permanent Position
② Institution Hired
③ Third-Party Labor Dispatch
④ Other

10. Have You Changed Employers Since Graduation?
(1) Yes. Please select the primary reasons for changing jobs (Select all that apply):
① Non-permanent position
② Unsatisfactory salary
③ No promotion pathway, limited growth potential
④ Job content unrelated to the major
⑤ Poor work environment and atmosphere
⑥ Job instability
⑦ Job too demanding, seeking a less strenuous position
⑧ Workplace too far from home
⑨ Other

(2) No

11. Are you considering changing jobs currently?
(1) Yes, please select the primary reasons for considering a job change (Select all that apply):
① Non-permanent position
② Unsatisfactory salary
③ No promotion pathway, limited growth potential
④ Job content unrelated to the major
⑤ Poor work environment and atmosphere
⑥ Job instability
⑦ Job too demanding, seeking a less strenuous position

approved by the Institutional Review Board at Fujian Health College

⑧ Workplace too far from home
⑨ Other

(2) No

12. Have you passed the Public Health Practitioner Assistant Physician Licensing Examination?
① Haven’t taken the exam yet, reason: ___
② Took the exam but didn’t pass the skills test
③ Passed the skills test, preparing for the theory exam
④ Already passed

13. Have you obtained any other professional qualifications or certifications?
① Health Manager
② Public Nutritionist
③ Infant Care Worker
④ Elderly Care Worker
⑤ Social Psychological Services (1+X Certification)
⑥ Other
⑦ None

14. Which of the following professional certifications do you think would be most helpful to you?
① Health Manager
② Public Nutritionist
③ Childcare Practitioner
④ Geriatric Caregiver
⑤ Social Psychological Services (1+X Certification)
⑥ Other

15. Please evaluate whether the medical college you graduated from provided sufficient training in the following professional modules and skills to meet the requirements of your job position. Five Levels of skills meet the job requirements: ⑤ very adequate ④ adequate ③ essentially adequate ② not adequate ① extremely inadequate.

Dimension	Course or Skill	Levels of Skills Meet the Job Requirements
Personal Qualities	Moral qualities	
	Ideals and beliefs	
	Life education	
	Professional competence	
	Humanistic literacy	
	Comprehensive judgment ability	
	Psychological quality	
	Innovation awareness	

approved by the Institutional Review Board at Fujian Health College

	Teamwork awareness<br>Safety awareness
**Fundamental Courses**	Medical Ethics<br>Computer Applications in Healthcare<br>Microbiology and Immunology<br>Physiology<br>Pharmacology<br>Biochemistry<br>Psychology
**Clinical Medicine Courses**	Diagnostic Medicine<br>Internal Medicine<br>Surgery<br>Obstetrics and Gynecology<br>Pediatrics<br>Emergency Medicine<br>Infectious Diseases
**Specialized Courses**	Epidemiology<br>Biostatistics<br>Occupational Health and Occupational Medicine<br>Environmental Health<br>Nutrition and Food Hygiene<br>Health Education and Promotion<br>Maternal, Child, and Adolescent Health<br>Basic Public Health Service Techniques<br>Social Medicine<br>Health Laws and Regulations<br>Integrated Preventive Medicine Skills Practicum
**Professional Skills**	Air Quality Testing Skill<br>Water Quality Testing Skill<br>Occupational Health Testing Skill<br>Food Hygiene Testing Skill<br>Child and Adolescent Health Assessment Skill<br>Epidemiological Methods and Skills<br>Statistical Methods and Skills<br>Health Education Skills<br>Basic Clinical Skills<br>Information Literacy and Professional Resource Use<br>Essential Computer Skills

approved by the Institutional Review Board at Fujian Health College

Literature Search and Review Skills

Scientific Writing and Research Skills

Teamwork and Collaboration Skills

Organizational and Coordination Skills

Communication and Counseling Skills

Community Mobilization Skills

16. Which knowledge or skills gained from the preventive medicine program do you think have been most helpful in performing your job?

17. What additional knowledge or skills do you think should be acquired or improved to better perform your job?

This questionnaire is now complete. Thank you very much for your participation!

approved by the Institutional Review Board at Fujian Health College
