# Supplementary material for: Effect of a three-years preventive medicine vocational education program on county-level healthcare workforce development in China: a cross-sectional study
Source: BMC Med Educ. 2025 Apr 11;25:522. doi: 10.1186/s12909-025-07095-w (PMC11992889; doi:10.1186/s12909-025-07095-w)
Supplement: Supplementary file 4 — Supplementary Material 4 [file 12909_2025_7095_MOESM4_ESM.pdf]

Supplementary Materials

Table1 General Curriculum of the Three-year Preventive Medicine Vocational Education Program

Dimension	Course or Skill	offering General Semester
**Personal Qualities**	Moral Qualities	First-year first-semester
	Ideals and Beliefs	First-year first-semester
	Life Education	First-year first-semester
	Professional Competence	From the first year to the third year
	Humanistic Literacy	First-year first-semester
	Comprehensive Judgment Ability	From the first year to the third year
	Psychological Quality	First-year first-semester
	Innovation Awareness	From the first year to the third year
	Teamwork Awareness	From the first year to the third year
	Safety Awareness	From the first year to the third year
**Foundamental Courses**	Medical Ethics	First-year second-semester
	Computer Applications in Healthcare	First-year second-semester
	Microbiology and Immunology	First-year second-semester
	Physiology	First-year second-semester
	Pharmacology	First-year second-semester
	Biochemistry	First-year second-semester
	Psychology	First-year second-semester
**Clinical Medicine Courses**	Diagnostic Medicine	First-year second-semester
	Internal Medicine	Second-year first-semester
	Surgery	Second-year first-semester
	Obstetrics and Gynecology	Second-year first-semester

	Pediatrics	Second-year first-semester
	Emergency Medicine	Second-year first-semester
	Infectious Diseases	Second-year first-semester
**Specialized Courses**	Field Epidemiology	Second-year second-semester
	Health Statistics Practice	Second-year second-semester
	Occupational Health and Occupational Medicine	Second-year second-semester
	Environmental Health	Second-year second-semester
	Nutrition and Food Hygiene	Second-year second-semester
	Health Education and Health Promotion	Second-year second-semester
	Maternal and Child Health Care	Second-year second-semester
	Basic Public Health Service Techniques	Second-year second-semester
	Social Medicine	Second-year second-semester
	Health Laws and Regulations	Second-year second-semester
**Professional Skills**	Air Quality Testing Skill	Second-year second-semester
	Water Quality Testing Skill	Second-year second-semester
	Occupational Health Testing Skill	Second-year second-semester
	Food Hygiene Testing Skill	Second-year second-semester
	Child and Adolescent Health Assessment Skill	Second-year second-semester
	Epidemiological Methods and Skills	Second-year second-semester
	Statistical Methods and Skills	Second-year second-semester
	Health Education Skills	Second-year second-semester
	Basic Clinical Skills	Second-year first-semester
	Information Literacy and Professional Resource Use	From the first year to the third year
	Essential Computer Skills	From the first year to the third year
	Literature Search and Review Skills	From the first year to the third year

	Scientific Writing and Research Skills	From the first year to the third year
	Teamwork and Collaboration Skills	From the first year to the third year
	Organizational and Coordination Skills	From the first year to the third year
	Communication and Counseling Skills	From the first year to the third year
	Community Mobilization Skills	From the first year to the third year
**Internship**	Medical Institutions of Primary Health Care Systems	Third-year first-semester
	County-level Centers for Disease Control and Prevention	Third-year second-semester

Note: These courses information is mainly derived from the national vocational education teaching standard for the Preventive Medicine in China, and it was implemented starting February 11, 2025.

Available from: http://www.moe.gov.cn/s78/A07/zcs_ztzl/2017_zt06/17zt06_bznr/bznr_zyjyzyjxbz/gdzyjy_zk/zk_yywsdl/
